# Evaluation of postoperative ascites after somatostatin infusion following hepatectomy for hepatocellular carcinoma by laparotomy: a multicenter randomized double-blind controlled trial (SOMAPROTECT)

**DOI:** 10.1186/s12885-018-4667-0

**Published:** 2018-08-23

**Authors:** Kayvan Mohkam, Michel Rayar, Jean-Philippe Adam, Fabrice Muscari, Agnès Rode, Philippe Merle, Pierre Pradat, Stéphanie Bauler, Isabelle Delfour, Laurence Chiche, Christian Ducerf, Karim Boudjema, Mickaël Lesurtel, Christophe Laurent, Jean-Yves Mabrut

**Affiliations:** 1 Department of General Surgery and Liver Transplantation, Hospices Civils de Lyon, Croix-Rousse University Hospital, 103, Grande Rue de la Croix-Rousse, 69317 Lyon Cedex 04, France; 2 Ecole Doctorale EDISS 205, EMR 3738, Claude Bernard Lyon 1 University, Lyon, France; 3 Department of Digestive Surgery and Liver Transplantation, Hôpital Pontchaillou, Rennes, France; 4 Department of Digestive Surgery and Liver Transplantation, Hôpital Haut-Lévêque, Bordeaux, France; 5 Department of Digestive Surgery and Liver Transplantation, Hôpital Rangueil, Toulouse, France; 6 Department of interventional radiology, Hospices Civils de Lyon, Croix-Rousse University Hospital, Lyon, France; 7 Department of Hepatology, Hospices Civils de Lyon, Croix-Rousse University Hospital, Lyon, France; 8 Centre for clinical research, Hospices Civils de Lyon, Croix-Rousse University Hospital, Lyon, France; 9 Department of clinical pharmacy, Croix-Rousse University Hospital, Lyon, France

**Keywords:** Hepatocellular carcinoma, Surgery, Ascites, Omatostatin, Andomized controlled trial

## Abstract

**Background:**

The majority of patients undergoing hepatectomy for hepatocellular carcinoma (HCC) suffer from underlying liver disease and are exposed to the risk of postoperative ascites, which is favored by an imbalance between portal venous inflow and a diminished hepatic volume. Finding a reversible, non-invasive method for modulating the portal inflow would be of interest as it could be used temporarily during the early postoperative course. Somatostatin, a well-known drug already used in several indications, may limit the risk of postoperative ascites and liver failure by decreasing portal pressure after hepatectomy for HCC in patients with underlying liver disease. We aimed to evaluate the impact of somatostatin postoperative infusion on the incidence of ascites following hepatectomy by laparotomy for HCC in patients with underlying liver disease.

**Methods/design:**

The SOMAPROTECT study is a multicenter randomized double-blind placebo controlled phase III trial comparing two arms of patients with underlying liver disease undergoing hepatectomy for HCC by open approach. All patients will have primary abdominal drainage before closure. Patients in the experimental arm will receive a postoperative intravenous infusion of somatostatin during 6 days. Patients in the control group will receive a placebo infusion for the same duration. The primary endpoint will be the presence or absence of postoperative ascites occurring during the 90-day postoperative course, defined as ≥500 ml/24 h of fluid in the drains during at least 3 days or any ascites requiring an invasive procedure comprising percutaneous puncture or drainage. Secondary endpoints will be duration and total volume of ascites, postoperative 90-day mortality and morbidity, liver failure, acute renal failure, length of stay in intensive care unit and hospital stay. The total number of patients to be enrolled was calculated to be 152.

**Discussion:**

Postoperative ascites remains a major issue after hepatectomy for HCC as it is associated with increased morbidity, liver and renal failure, the need for specific treatments and prolonged hospital stay. This study represents the first randomized controlled trial to assess the benefits of somatostatin on the risk of postoperative ascites after surgery for HCC.

**Trial registration:**

NCT02799212 (ClinicalTrials.gov identifier). Registered prior to conducting the research on 9 June 2016.

## Background

Liver resections represent the best therapeutic option for resectable hepatocellular carcinomas (HCCs) in patients not eligible for liver transplantation and also as first-line treatment in some patients with a project for liver transplantation according to the French national policy of organ allocation [1], with a 50–70% 5-year overall survival [2]. The vast majority of patients developing HCC suffer from underlying liver disease, and despite the progress made in terms of perioperative management, patients undergoing hepatectomy for HCC are exposed to the risk of postoperative ascites and subsequent morbidity, which occurs in up to 56% of patients [3]. While the laparoscopic approach may decrease the rate of postoperative ascites after hepatectomy [4], a large proportion of liver resections for HCC are still made through a traditional open approach and therefore, postoperative ascites remains a major concern in that situation.

Postoperative ascites in patients undergoing hepatectomy is associated with an increased risk of overall postoperative morbidity and acute renal failure, the need for specific treatment (including albumin infusion, diuretics and abdominal puncture) and prolonged hospital stay [3]. It is favored by an imbalance between the portal venous inflow and the diminished hepatic venous outflow in the remnant liver, which may lead to portal hyperperfusion, acute portal hypertension and ascites [5]. This phenomenon is known as the small-for-size syndrome, which has been initially described after partial liver transplantation [6] but is also one of the main mechanisms involved in postoperative ascites after hepatectomy [5, 7, 8]. This risk is increased especially in patients undergoing hepatectomy for HCC, as the majority of patients with HCC suffer from underlying liver disease and subsequently, from low or mild portal hypertension.

In order to limit the risk of postoperative acute portal hypertension and ascites, surgical techniques of portal inflow modulation, such as splenic artery ligation, splenectomy and porto-systemic shunts have been proposed after partial liver transplantation [9], and also after extended liver resection in experimental animal models [10–12] and in patients with cirrhosis [13–15]. These techniques aim to divert the portal inflow and decrease portal pressure in order to avoid the small-for-size syndrome and postoperative ascites. However, these surgical methods of portal inflow modulation are invasive and may induce specific morbidity such as hemorrhage, portal thrombosis, asplenia and encephalopathy [16]. Additionally, all reported surgical techniques of portal inflow modulation are irreversible, while liver regeneration mainly concerns the 5 to 7 first days following resection [17, 18]. After that regeneration period, there is no more need for a decrease in portal flow, which might even induce encephalopathy or impair liver regeneration [19]. In this context, finding a reversible and non-invasive method for modulating the portal inflow would be of great interest, as it could be used temporarily during the early postoperative course to limit the risk of acute portal hypertension without having the drawbacks of a definitive and invasive surgical method of portal inflow modulation.

Somatostatin, a natural hormone with splanchnic vasoconstriction properties, is a valid therapy of upper gastro-intestinal bleeding as it decreases both portal flow and pressure in cirrhotic patients [20]. To date, there has been no study assessing the impact of somatostatin infusion on postoperative ascites following partial hepatectomy. However, a few experimental studies have demonstrated the efficacy of somatotsatin in improving the outcome of animals undergoing partial liver transplantation: For instance, Xu et al. demonstrated that somatostatin infusion decreased the portal pressure significantly in a rat model of partial liver transplantation [21]. Hessheimer et al. showed in a porcine model of partial liver transplantation that somatostatin infusion decreased the rate of postoperative ascites and improved the outcome of swine undergoing partial liver transplantation, not only by diminishing the portal pressure, but also by a direct cytoprotective mechanism [22]. In the clinical setting, Ozden et al. reported a single clinical case of post liver transplant small-for-size syndrome successfully treated by somatostatin infusion combined with beta blockers, after failure of splenic artery ligation [23]. Recently, our team assessed the intraoperative effects of somatostatin on splanchnic hemodynamics of swine and showed that somatostatin decreased portal flow and restored a normal hepatic venous pressure gradient in animals undergoing extended hepatectomy [24]. These findings together with somatostatin’s inherent properties suggest that this molecule may play a favorable role in the postoperative course of patients with underlying liver disease undergoing hepatectomy for HCC by open approach.

Therefore, we aimed to test through a multicenter randomized controlled trial the hypothesis that somatostatin infusion after hepatectomy by laparotomy for HCC would decrease the incidence of postoperative ascites.

## Methods/design

### Protocol overview

**Fig. 1:**
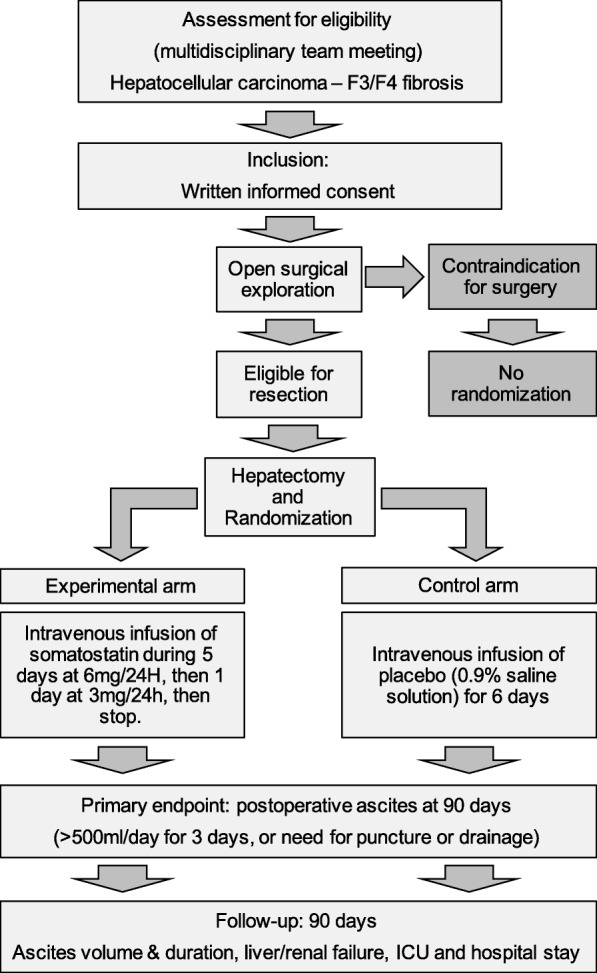
Study flow chart

The SOMAPROTECT study will consist of a multicenter randomized double-blind placebo controlled phase III trial comparing two groups of patients with underlying liver disease undergoing hepatectomy for HCC by open approach, who will receive postoperative infusion of somatostatin or placebo (Fig. 1). After therapy validation in a HCC dedicated oncological multidisciplinary team meeting, patients with a resectable HCC and with underlying liver disease who are eligible for a liver resection by open approach will be considered for inclusion in the study.

In both arms of the study, patients will undergo a hepatectomy by open approach, with intraoperative measurement of portal vein and inferior vena cava pressures before and after liver resection. All patients will benefit from an abdominal drainage. Randomization will be performed after the abdomen is opened and any contraindication for resection (i.e. peritoneal carcinomatosis) has been precluded. Patients will be randomized in equal proportions in two groups. Patients in the experimental group will receive an intravenous postoperative infusion of somatostatin (Eumedica SA, Manage, Belgium) during 5 days at 6 mg/day, followed by one day at 3 mg/day. Treatment will be stopped at the end of day 6. Patients in the control group will receive a placebo infusion consisting of a daily 50 ml intravenous infusion of 0.9% NaCl during 6 days. No additional postoperative infusion or injection of somatostatin or any analogue of somatostatin will be performed during postoperative course.

In order to allow a double-blind design, preparation of somatostatin or placebo will be performed outside the ward where the patient is located by a pharmacist aware of the randomization results. The surgeons and nurses in charge will thus be unaware of the allocated treatment. Intraoperative management of vascular fluids and postoperative management of ascites will be defined by a common protocol. Abdominal drains will be left in place during at least 4 days and will be mobilized systematically from postoperative day 5 onwards.

### Inclusion criteria

The study will include patients with HCC who are eligible for open hepatic resection. The inclusion criteria are as follows: patient age ≥ 18 years; patients with HCC diagnosed by histology or by imaging findings according to the definition of Barcelona Clinic Liver Cancer Group [25]; patients with a single or multiple HCCs deemed to be resectable with a curative intent at the preoperative evaluation; patients for whom an indication for hepatectomy by open approach (laparotomy) has been decided and approved by a multidisciplinary team meeting; patients with any underlying liver disease with or without proven cirrhosis, regarding histological features (including F3-F4 fibrosis with or without cirrhosis) or with other evidence of a diseased liver if no biopsy has been performed preoperatively (dysmorphic liver or evidence of portal hypertension at imaging findings, oesophageal varices at endoscopy); patients with ability to understand and sign a written informed consent form; patients who will be available for follow-up.

### Exclusion criteria

All patients who do not meet all the inclusion criteria will not be included. The other non-inclusion criteria are the common contraindications for surgery related to patient status, disease extension and operative technique. Patient-associated non-inclusion criteria will include: Patients with any other simultaneous experimental treatment and those with evidence of a healthy liver at biopsy (i.e. F0, F1 or F2). Disease-associated non-inclusion criteria will include: another histologic type of hepatic tumor besides HCC; presence of distant extra-hepatic metastases, including peritoneal carcinomatosis; existence of complete portal thrombosis of the main portal trunk. Patients with an indication for laparoscopic approach will also be excluded.

### Endpoints and outcome measurements

The primary endpoint will be the presence or absence of postoperative ascites occurring during the postoperative course (within 90 postoperative days). Postoperative ascites will be defined by the existence of ≥500 ml / 24 h of fluid in the drains during at least 3 days, or any ascites requiring an invasive procedure comprising percutaneous puncture or drainage (radiologically or surgically). This definition of ascites of ≥500 ml/24 h is the most common definition of postoperative ascites in the medical literature: several widely reported studies have used that definition [26–31]. Moreover, Azoulay et al. added to this definition the necessity of a 3-day period, which avoids selection bias related to postoperative fluid caused by bleeding, residual lavage or bile leakage [27]. In addition to this definition, we added the need for an invasive procedure as a definition of ascites because some patients may develop ascites after drain removal, or may develop some ascites that is not drained appropriately by the surgical drain, which will require additional invasive management such as puncture or drainage.

Secondary endpoints are duration and total amount of ascites, postoperative overall 90-day morbidity, the existence and the severity of postoperative liver failure, the existence and the severity of postoperative acute renal failure, postoperative mortality (overall and liver-related), length of stay in intensive care unit, and length of hospital stay. A standardized definition and/or grading system will be considered for all these secondary endpoints as follows: duration of ascites will be defined as the number of days with ascites ≥500 ml/24 h; total amount of ascites will be defined as the total quantity of ascites collected by the drains from surgery to drain removal, associated with the total quantity of ascites collected by radiological puncture or drains if secondary drainage or puncture has been performed; postoperative morbidity will be graded according to the Dindo-Clavien scale [32]; postoperative liver failure will be defined and graded according to the International Study Group of Liver Surgery (ISGLS) definition and grading system [33]; postoperative acute renal failure will be defined according to the “Acute Dialysis Quality Initiative” (ADQI) definition and graded according to the “Risk, Injury, Failure, Loss, End-stage kidney disease” (RIFLE) Scale [34]; postoperative mortality will be defined as any death occurring within 90 postoperative days. Cases of death will be divided into liver failure-related versus liver failure-unrelated deaths.

### Randomization

Patients will be randomized during the operative procedure after the abdomen has been opened and the peritoneal cavity explored, precluding any contraindication for surgical resection. For patients in the experimental arm, an intravenous infusion of somatostatin (Eumedica SA, Manage, Belgium) at full-dose (6 mg/24 h) will be started at abdominal closure, and performed during 5 days, followed by a half-dose (3 mg/24 h) infusion during 1 day before it is stopped. Patients in the control arm will receive placebo infusion consisting of 50 ml intravenous infusion of 0.9% NaCl during 6 days.

The randomization will be performed using the stratified block randomization method for each center. A randomization list will be generated for each center and envelopes will be prepared and blinded for allocation during surgery according to consecutive inclusion.

### Preoperative work-up

Patients eligible for study will undergo complete conventional preoperative work-up, including physical examination, standard blood test (comprising hemostasis tests, platelet count, indocyanin green clearance test, dosage of liver enzymes, alpha-fetoprotein, serum creatinine concentration), oesogastroscopy for evaluation of oesophageal and/or gastric varices, thoraco-abdomino-pelvic computed tomography (CT), to assess resectability and search for extrahepatic metastasis, portal thrombosis, other signs of portal hypertension (venous collaterals, splenomegaly), tumor features will be precisely evaluated by radiologists regarding size, number, the Milan Criteria [35] and the Alpha score [36]. Evaluation of the cardiac function is optional and left at the anesthesiologist’s discretion regarding patient background. All patient files will be discussed at a multidisciplinary staff, and the indication for hepatectomy by open approach will be validated before any consideration for patient inclusion.

### Treatment methods

#### Global preoperative care

Administration of immunonutrition (Oral Impact®, Nestlé) will be performed in all patients 7 days before surgery. Cessation of alcohol drinking will be requested at least one month before surgery.

### Anaesthetic technique

Premedication will be administered at the team’s discretion. Patients will receive prophylactic antibiotics 30 min prior to the abdominal incision. Standard monitoring will be performed using 2 peripheral venous catheters, while the placement of a central venous catheter and/or an arterial pressure monitoring catheter is not mandatory and left at the anesthesiologist’s discretion. No epidural analgesia will be used as patients with underlying liver disease may exhibit hemostatic disorders, thus increasing the risk of epidural bleedings.

Induction of general anesthesia will be performed with intravenous injection of propofol, remifentanil, and Cis-astracurium. Maintenance of general anesthesia will be performed with halogen gas, remifentanil and myorelaxation will be obtained with intravenous boluses of cis-astracurium. Ventilation will be performed with a 6 to 8 ml/kg tidal volume, a positive end-expiratory pressure around 5 cmH_2_O, and the respiration frequency will be adjusted to maintain end-tidal CO^2^ between 30 and 35 mmHg. Regarding the intraoperative conditions, the positive end-expiratory pressure could be modified or even interrupted at anesthesiologist’s discretion.

Administration of intravenous fluids will be performed appropriately regarding the cardio-circulatory monitoring with a regimen of 4 to 6 ml/kg/h of isotonic crystalloid solution, at the anesthesiologist’s discretion. Packed red blood cell transfusion will be performed in case of blood loss > 1500 ml or anemia < 7 g/dl (or < 10 g/dl in case of coronary disease). No prophylactic antifibrinolytic agent will be used systematically.

### Surgical technique

All procedures will be performed by open approach, and will begin with a careful exploration of the abdominal cavity to preclude any contraindication for resection. The type of incision, the parenchymal transection technique, and the choice of portal clamping will be left at the surgeon’s discretion.

### Intraoperative measurement of hepatic venous pressure gradient

An intraoperative measurement of the hepatic venous pressure gradient will be performed after hepatectomy and after hemodynamic stability is obtained. A direct puncture of the portal vein followed by direct puncture of the infra hepatic inferior vena cava will be performed, using a 0.5 mm trocar connected to an arterial pressure monitoring catheter, which itself will be connected to a cardio-tensional scope, thus displaying the pressure value. The hepatic venous pressure gradient will be calculated with the following formula (*Hepatic venous pressure gradient = Portal venous pressure – infrahepatic inferior vena cava pressure*).

### End of the surgical procedure

At the end of each procedure, a surgical drain will be placed within the surgical site in order to drain the abdominal cavity and potential postoperative fluids. The modality of drainage utilized will be standardized in each center and will consist of the use of a closed-circuit drain without suction.

### Somatostatin or placebo administration

The treatment is started after abdominal closure and stopped after 6 days in both arms. No postoperative administration of somatostatin or any somatostatin analog will be performed postoperatively. The administration of placebo will not induce any significant changes in the management of patients, given all patients undergoing hepatectomy have intravenous treatments during this period.

### Postoperative care

#### Global postoperative care

After hepatectomy, the patient will stay in an intensive care unit during a short period of 2 to 4 days (for information purposes only, the duration is left at the discretion of the team in charge of the patient) or will return directly to the conventional hospitalization unit. Besides the postoperative infusion of somatostatin (experimental arm) or placebo (control arm), all other postoperative therapies will be standardized for all patients regardless of the arm in which patients are included. In addition, patients will benefit from a standard postoperative monitoring including physical examination (including umbilical circumference, weight, heart rate, arterial pressure, urine out-put) twice a day, standard liver tests and renal blood tests at postoperative day 1, then every 48 h until postoperative day 15 (postoperative days 3, 5, 7, 9, 11, 13, 15) or patient discharge. The tested blood parameters must include: INR, prothrombin time, serum total bilirubin, SGOT, SGPT, and creatinine. Other blood tests may be performed at the physician’s discretion. The glomerular filtration rate will be estimated according to the CKD-EPI formula. A computed tomography will be performed approximately one month after the procedure (+/− 5 days), to assess the presence of subclinical ascites or other intra-abdominal cavity complications.

### Abdominal drain

All patients will benefit from the placement of a surgical drain during the operative procedure. All drains will be left in place during 4 days without any mobilization. Drain mobilization will start at postoperative day 5. The total amount of fluid in the drains will be monitored every 8 h until drain removal.

### Management of postoperative ascites

The management of postoperative ascites will be standardized according to the International Ascites Club’s recommendations [37] and will be the same for all patients in each participating center No systematic fluid restriction will be performed. Water drinking will be allowed at postoperative day 1, while oral feeding will be authorized once bowel activity has returned. Administration of 1 l/day of intravenous fluids will be performed until day 2, then stopped. The type of fluid administered will be crystalloid isotonic solution. In case of postoperative ascites development, the standardized treatment will associate:

- Water restriction (1 l/day) and salt restriction
- 20% albumin: 200 ml per day + 100 ml for every 3 l of drained ascites
- Administration of diuretics:
  - Spironolactone, starting at 50 mg/day; increased from 25 mg every 48 h in the absence of any decrease in ascites, with a maximum dosage of 200 mg
  - In the absence of acute renal failure, furosemide will be associated to spironolactone at a dosage of 20 mg/day, increased to 40 mg/day after 48 h in the absence of any decrease in ascites
  - Diuretics will be administered orally or intravenously in case of intestinal ileus.

This specific treatment of ascites will be discontinued 48 h after ascites resolution.

In case of evidence of intraabdominal infection or tense ascites as defined by the International Ascites Club [37], an invasive procedure will be performed to evacuate the intraabdominal fluid. The choice between puncture, radiological or surgical drainage is left at the physician’s discretion. In all other clinical situations, invasive procedures will be avoided in first intent.

### Management of postoperative acute renal failure

A major attention will be paid to depict acute renal failure during the postoperative course, as it is associated with a significant increase in postoperative morbidity, ranging from 5 to 15% [38]. In case of acute renal failure, a stage-specific treatment will be performed based on the RIFLE (Risk, Injury, Failure, Loss, End-stage) scale, as follows:

- *In case of “risk” of acute renal failure (R, increase of creatinine by a ratio of 1.5, or 25% decrease of glomerular filtration rate, or urine out-put below 0.5 ml/kg/h during 6 h),* the management includes an initial decrease in diuretics dosage (or discontinuation if minimum dosage were used), followed by complete discontinuation if no improvement occurs after 48 h.
- *In case of “injury” of the kidney (I, increase of creatinine by a ratio of 2, or a 50% decrease of glomerular filtration rate, or urine out-put below 0.5 ml/kg/h during 12 h),* the management includes discontinuation of diuretics, followed by intravenous administration of 1000 ml/day of crystalloid fluids (and albumin [60 g/day] in case of postoperative ascites) if no improvement occurs after 48 h.
- *In case of “failure” of the kidney (F, increase of creatinine by a ratio of 2, or a 50% decrease of glomerular filtration rate, or urine out-put below 0.5 ml/kg/h during 12 h),* the management includes discontinuation of diuretics combined with intravenous administration of 1000 ml/day of crystalloid fluids (and albumin [60 g/day] in case of postoperative ascites).

### Surgical specimen analysis

Histological analysis of the specimen will include tumor grade according to Edmondson’s scale, tumor size, tumor number, margins, radicality of resection (R0, R1, R2), the existence of portal microthrombosis, the existence of vascular emboles, the existence of perineural invasion, the existence of fibrosis in the adjacent non-tumorous liver parenchyma (F3, F4), the existence of a rupture of the Glisson’s capsule in case of peripheral tumors.

### Follow-up

The inclusion period will last 24 months, allowing the inclusion of 152 patients over that period. The project will finish 90 days after the end of the inclusion period, once the “end of research visit” of the last included patient has been completed. The total study period will therefore correspond to a 27-month period. Two follow-up visits will be scheduled after hospital discharge: at 1 month +/− 7 days (M1) after the surgery, and at 3 months +/− 14 days (M3).

The M1 follow-up visit is performed by the surgeon in charge of the patient and will comprise standard physical examination (including wound examination, measurement of weight and umbilical circumference, heart rate, arterial pressure), biological analyses (INR, prothrombin time, serum total bilirubin, SGOT, SGPT, and creatinine) and a contrast-enhanced abdomino-pelvic CT to asses the existence of ascites and to depict any other postoperative complication. This postoperative 1-month CT scan is in accordance with the standard of care of all five centers involved in the present study, and should not be considered as part of the experiment but as part of the standard follow-up management.

The M3 follow-up visit is performed by either the surgeon or the oncologist/hepatologist in charge of the patient. This visit is also considered as the “end of research visit” and will comprise the same examinations than those in M1.

### Participating centers

All participating centers are required to be experienced in hepatic surgery and management of HCC. Overall, 4 French centers will participate in the study: The Croix-Rousse University Hospital in Lyon, the Pontchaillou University Hospital in Rennes, the Haut-Lévêque University Hospital in Bordeaux, and the Rangueil University Hospital in Toulouse.

### Sample size and statistical analysis

The hypothesis of this phase III study is that the postoperative intravenous infusion of somatostatin during the 6 first postoperative days will reduce the rate of patients developing postoperative ascites. Based on previous published studies, the incidence of postoperative ascites in patients undergoing hepatectomy for HCC is estimated to be 35% (the reported incidence in literature varies between 5 and 56% [26–31], with a median value of 35%, which is consistent with our own experience of 33% postoperative ascites following hepatectomy by laparotomy for HCC at our institution [unpublished data]).

We postulate that we will observe a reduction of the incidence of postoperative ascites from 35% in the control arm to 15% in the experimental arm. In order to demonstrate a 20% difference with an alpha risk of 5% and a power of 80% using a two-tailed hypothesis, 72 patients in each arm will be necessary. Due to the short follow-up period of 90 days in this study, we assume that no more than 1 patient will be lost to follow-up. Additionally, we assume that 5% (*n* = 7) of the included patients will be precluded from surgical resection due to the findings of the surgical exploration (for instance, because of peritoneal carcinomatosis at surgical exploration), and thus will not be evaluable. Therefore, a total of 152 patients will have to be included in this study. To reach this number of patients, a 2-year inclusion period will be necessary.

Patients included in this study will be analyzed on an intent-to-treat basis, and therefore kept in their initial treatment arm, even if the experimental treatment must be interrupted for any reason. The primary endpoint will be expressed in total number of cases in each group and in percentages with 95%-confidence interval. The secondary endpoints’ categorical variables (severity of ascites, postoperative morbidity, postoperative liver failure, postoperative acute renal failure, postoperative mortality) will also be expressed in total number of cases in each group and in percentages with 95%-confidence interval. These categorical variables will be compared between the two study arms using the chi-2 test or the Fisher’s exact test, as appropriate. The secondary endpoints’ continuous variables (duration of ascites, total amount of ascites, length of stay in intensive care unit, length of hospital stay) will be expressed as median values and range, and will be compared using the Mann-Whitney U-test. In order to take account of the potential effects of confounders on the primary endpoint, univariate and multivariate logistic regressions will be performed.

### Ethical considerations and written informed consent

The present study protocol was approved by the Institutional Review Board, the French National Agency for Medicines and Health on the 11 of July 2017 under the registration number 170335A-42, and the “Est II” ethic committee on the 11 of September 2017, under the registration number EudraCT2016–004230-20. The institutional promoter is the University Hospital of Lyon (Hospices Civils de Lyon), France. The trial has been registered on the ClincialTrials.gov website under the identification number NCT02799212. This study received a grant from the French National Cancer Institute in January 2016 (Grant reference number: PHRC-K15–103).

A written informed consent will be obtained from each participating patient before inclusion to the study. The appropriate time to inform the patient about the study (before or after the board meeting) is left to the medical team’s discretion. The patient will be given a minimum period of 1 week to consider the project before signing the written inform consent. The written inform consent form signed by the patient is obtained by the local investigator the day prior to surgery as patients are hospitalized at least one day before surgery. The head of each team will be the local investigator of each participating center and will be responsible for patients’ information, inclusion and follow-up. Each investigator shall undertake to fulfill the obligations of the law and to conduct research according to Good Clinical Practice guidelines, in accordance with the terms of the Declaration of Helsinki.

## Discussion

Surgical resection represents the only chance for cure for most patients presenting with early stage HCC and not eligible for liver transplantation. Unfortunately, most patients developing HCC also suffer from underlying liver disease, which exposes them to the risk of postoperative ascites and subsequent severe morbidity. Currently, the means to prevent postoperative ascites and post hepatectomy liver failure are mainly prophylactic, including preoperative management of diabetes, malnutrition, cardiorespiratory disease, preoperative optimization of the future liver remnant by contralateral portal vein embolization and biliary drainage in case of cholestasis. In addition to those prophylactic means, some authors have proposed to perform concomitant surgical portal inflow modulation in association to hepatectomy, either by splenic artery ligation or splenectomy [13–15, 39], but those techniques are invasive, irreversible and quite risky, especially in patients with underlying liver disease.

Somatostatin is a well-known drug already used in several indications, including pancreatic fistula, upper gastrointestinal bleeding, acromegaly, and endocrine tumors, with considerable long-term experience. Treatment dosage and duration that may be used in the present study are the same than those already used in upper gastrointestinal hemorrhage related to variceal bleeding, as this configuration allows a significant decrease in portal pressure [20].

The present study represents the first randomized controlled trial to assess the benefits of somatostatin on the risk of postoperative ascites after surgery for HCC. If the efficacy of somatostatin in preventing postoperative ascites is demonstrated, it would represent a novel and original therapy that would increase the safety of hepatectomy for HCC in patients with underlying liver disease in a context where hepatectomy remains the best curative therapeutic option in patients unsuitable for liver transplantation.

## Abbreviations

ADQI: Acute dialysis quality initiative
CT: Computed tomography
HCC: Hepatocellular carcinoma
ISGLS: International study group on liver surgery
RIFLE: Risk, injury, failure, loss, end-stage;
